# Mental Aberration, Consequent upon Pelvic Disease

**Published:** 1901-04

**Authors:** Chas. Wilson

**Affiliations:** Interne


### Clinical Report.

---

### MENTAL ABERRATION, CONSEQUENT UPON PELVIC DISEASE.

By Dr. L. G. HANLEY,
Gynecologist, Buffalo Hospital of the Sisters of Charity.

Reported by CHAS. WILSON, M. D., Interne.

MRS. R., aged 25, admitted to hospital July 18, 1900; married 9 years; two children; youngest child aged 6 years. Age of first menstruation, 13. Has been troubled with dysmenorrhea since her arrival at puberty. She was brought to the hospital suffering with severe pain in head. Complained also of severe pain in back and abdomen; height, 5 feet 4 inches; greatly emaciated and anemic; is greatly depressed and would sit for hours staring into space and melancholic. During menstrual period becomes violent, and always feared that she may do harm to herself or children. Says she likes to be alone, but afraid that something terrible will happen if left by herself.

Examination shows a laceration of cervix, retroflexion of uterus, with an enlargement of both ovaries. Patient prepared for abdominal section and trachelorrhaphy. When the abdomen was opened the uterus was found to be bound down posteriorly and each ovary about the size of a small orange and of a hematomatous nature. Both ovaries were removed and uterus anchored to anterior abdominal wall. The bilateral laceration of cervix repaired. Patient remained in hospital thirty-two days, during which time she displayed no evidences of uneasiness, mental or physical, beyond the ordinary. She was allowed to go home and has returned at intervals of three weeks. She says at present time (March 11, 1901) that she feels perfectly well, sleeps well, which for four years she has been unable to do excepting in a disturbed manner; that she weighs 140 pounds and with the exception of slight attacks of flashes of heat and cold, which come on about once a day, is perfectly well.

There seems to be no doubt but that the mental psychosis in this case was due in great part to the condition of pelvic disease, and that return to normal mental condition was due to operative interference.

